# A Systematic Review of Psychosocial Interventions to Cancer Caregivers

**DOI:** 10.3389/fpsyg.2017.00834

**Published:** 2017-05-23

**Authors:** Fang Fu, Huaijuan Zhao, Feng Tong, Iris Chi

**Affiliations:** ^1^Department of Social Work, Fudan UniversityShanghai, China; ^2^Anhui Normal UniversityWuhu, China; ^3^Sichuan International Studies UniversityChongqing, China; ^4^University of Southern CaliforniaLos Angeles, CA, United States

**Keywords:** psychosocial intervention, cancer caregiver, systematic review, effectiveness of intervention, depression, anxiety, quality of life

## Abstract

**Objective:** To systematically review the effect of psychosocial interventions on improving QoL, depression and anxiety of cancer caregivers.

**Methods:** We conducted a systematic review of psychosocial interventions among adult cancer caregivers published from 2011 to 2016. PsycINFO, PubMed, Proquest, Cochrane Library, Embase, Applied Social Sciences Index and Abstracts (ASSIA), Cumulative Index to Nursing and Allied Health Literature, Social Sciences Citation Index (SSCI) and EBSCO, China National Knowledge Infrastructure (CNKI) and WANFANG were searched. Inclusion criteria were: randomized controlled trails (RCTs); psychosocial intervention to cancer caregivers; psychosocial health indicators including quality of life, depression or anxiety.

**Results:** 21 studies out of 4,666 identified abstracts met inclusion criteria, including 19 RCTs. The intervention modes fell into the following nine categories: family connect intervention, self-determination theory-based intervention (SDT), cognitive behavioral therapy (CBT), emotion-focused therapy (EFT), comprehensive health enhancement support system (CHESS), FOCUS programme, existential behavioral therapy (EBT), telephone interpersonal counseling (TIP-C), problem-solving intervention (COPE).

**Conclusion:** paired-intervention targeting self-care and interpersonal connections of caregivers and symptom management of patients is effective in improving quality of life and alleviating depression of cancer caregivers while music therapy is helpful for reducing anxiety of cancer caregivers.

## Introduction

Today, the morbidity of cancer in China is on the climb, and the disease has become a widespread social problem. According to the 2012 Chinese Cancer Registry Annual Report released by the Chinese Cancer Registry Center in 2013, every year there are 3.12 million new cases of malignant tumors and over 2 million deaths (Ye and Zhu, 2005). Every day 8,550 people receive a confirmed diagnosis, and about six people are diagnosed with cancer every single minute (World Health Organization, 2015). A tumor is a severe stressful event for both patients and caregivers, while caregivers are faced with heavier and more complicated pressures (Ke et al., 2007). First of all, caregivers suffer long-term anxiety and fear about the death of their loved one (Li, 2014). Secondly, after the diagnosis is made, caregivers have to take the responsibility of plenty of activities without any preparation (Yabroff and Kim, 2009). What's more, the huge expense of cancer treatment also brings heavy economic burden to caregivers (Xia et al., 2014). Furthermore, caregivers social intercourse and work can also be influenced by taking care of the patients. Therefore, cancer caregivers suffer pressure from biological, psychological, economic and social aspects.

However, the needs of caregivers are often neglected and they are more likely to suffer psychological problems and become “invisible patients” (Xia et al., 2014). Empirical evidence also showed that the issue of depression and anxiety among cancer caregivers is more significant than that of patients. Studies indicate that the incidence rate of depression among cancer caregivers lies between 12 and 59%, and the incidence rate of anxiety is between 30 and 50% (Grunfeld et al., 2004), while the rates of depression and anxiety among cancer patients are between 10–25% and 19–34%, respectively (Li et al., 2000). The research of Ye and Zhu (2005) points out that 53.8% of cancer caregivers suffer minor or moderate depression, while 56.9% of them show symptoms of anxiety.

At present, there is a growing number of studies on psychosocial interventions to cancer caregivers, but there are relatively few reviews on the efficacy of different modes of interventions. One review was conducted many years ago, and the evidence was collected before 2001 (Schildmann and Higginson, 2010). Another study only reviewed psycho-educational interventions for cancer caregivers and focused on comparing the merits and demerits of different intervention designs (Schildmann and Higginson, 2010). Two recent systematic reviews emphasized intervention studies before 2011, and one of them only examined intervention effects of different means of interventions on single outcome variable. For instance, Waldron et al. (2013) made systematic reviews of the efficacy of different modes of psychosocial interventions in enhancing the quality of life for cancer caregivers. The systematic review by Applebaum and Breitbart (2013) was relatively thorough. It concluded psychosocial interventions performed before 2011 by informal caregivers including relatives, friends and spouses of cancer patients, and investigated the efficacy of psychological education, problem-solving, supportive therapy, home care, cognitive behavioral therapy (CBT), interpersonal therapy, complementary and alternative medicine interventions, existential therapy and other means of intervention in alleviating the burden of cancer caregivers (Applebaum and Breitbart, 2013).

Since 2011, progress has been achieved in studies concerning cancer caregivers. Firstly, psychosocial interventions to caregivers are not just confined to one single outcome variable. Instead, more than two outcome variables are taken into consideration. For example, there are studies that carry out interventions directed on the quality of life, depression and anxiety of caregivers (Hendrix et al., 2013) or on other variables such as self-efficacy and self-confidence (Collinge et al., 2013). Secondly, more and more studies have collected follow-up data after the interventions to examine the long-term effect (Sherwood et al., 2012; Boele et al., 2013). Furthermore, a growing number of randomized control groups no longer adopt a no-intervention policy when it comes to dealing with control groups. Instead, they resort to an active approach toward implementing controls, such as providing disease-related or care-related information, and discussing the disease in a supportive environment (Badger et al., 2011; Porter et al., 2011; Hendrix et al., 2013). More and more randomized controlled trials (RCT) have now been applied to psychosocial interventions to cancer caregivers, and the quality of intervention studies has been improved. Therefore, the purpose of the present review is to review the effectiveness of psychosocial interventions for cancer caregivers and provide concrete support for promoting the quality of life of them.

## Methods

### Search strategy

Publications from 2011 to 2016 were systematically selected. A literature search was conducted in the following digital databases: PsycINFO, PubMed, Proquest, Cochrane Library, Embase, Applied Social Sciences Index and Abstracts (ASSIA), Cumulative Index to Nursing and Allied Health Literature, Social Sciences Citation Index (SSCI) and EBSCO, China National Knowledge Infrastructure (CNKI) and WANFANG. Search words were the following: cancer/tumor, family/spouse/partner, caregiver/carers, psychosocial intervention/treatment/therapy, clinical trial/explanatory trial/pragmatic trial/randomized controlled trial/RCT. Search terms were the following: (cancer/tumor) AND (caregiver/carers/family/spouse/partners) AND (psychosocial intervention OR treatment/therapy) AND (clinical trial/explanatory trial OR pragmatic trial OR randomized controlled trial OR RCT), and search terms in different databases were slightly different.

### Selection strategy

A pair of independent raters selected abstracts for full review based on inclusion/exclusion criteria. Primary researcher resolved any discrepancies and produced final list of studied for full-text review.

All papers selected for final inclusion met the following criteria: (i) employed a psychosocial intervention aimed specifically to target cancer caregivers needs without a primary focus on the cancer patient; (ii) psychosocial health indicators including quality of life, depression or anxiety; (iii) RCT method adopted; (iv) written in Chinese or English; (v) publication of literature between 2011 and 2016.

### Review strategy

A pair of raters reviewed relevant studies and extracted data respectively. The discrepancies were resolved by primary researcher and final data were entered into a database management system. In consideration of the heterogeneity of interventions and data, this research adopts Crocharane as the risk of bias (ROB) assessment tool to evaluate the overall quality of the research. The assessment includes six perspectives: selection bias (selected by random sequence or allocation concealment), performance bias (blinding of participants and personnel), detection bias (blinding of outcome assessment), attrition bias (incomplete outcome data), reporting bias (comprehensive reporting) and other bias. Then, we scored every work according to these six aspects, with 2 points for “yes,” 1 point for “unknown” and 0 point for “no.” The total score was divided into three types, which stand for different levels of overall ROB: 0–4 points are for high risks, 5–8 points medium risks, and 9–12 points low risks. The report of systematic reviews is standardized by PRISMA (Preferred Reporting Items for Systematic Reviews and Meta-Analyses).

### Statistical analysis

Descriptive and explanatory data analyses were presented for a general picture of psychosocial intervention for cancer caregivers from 2011 and 2016. Effective size (Cohen d) were analyzed for each study measuring the difference in outcomes of quality of life, depression and anxiety when available data permitted.

## Results

**Figure 1:**
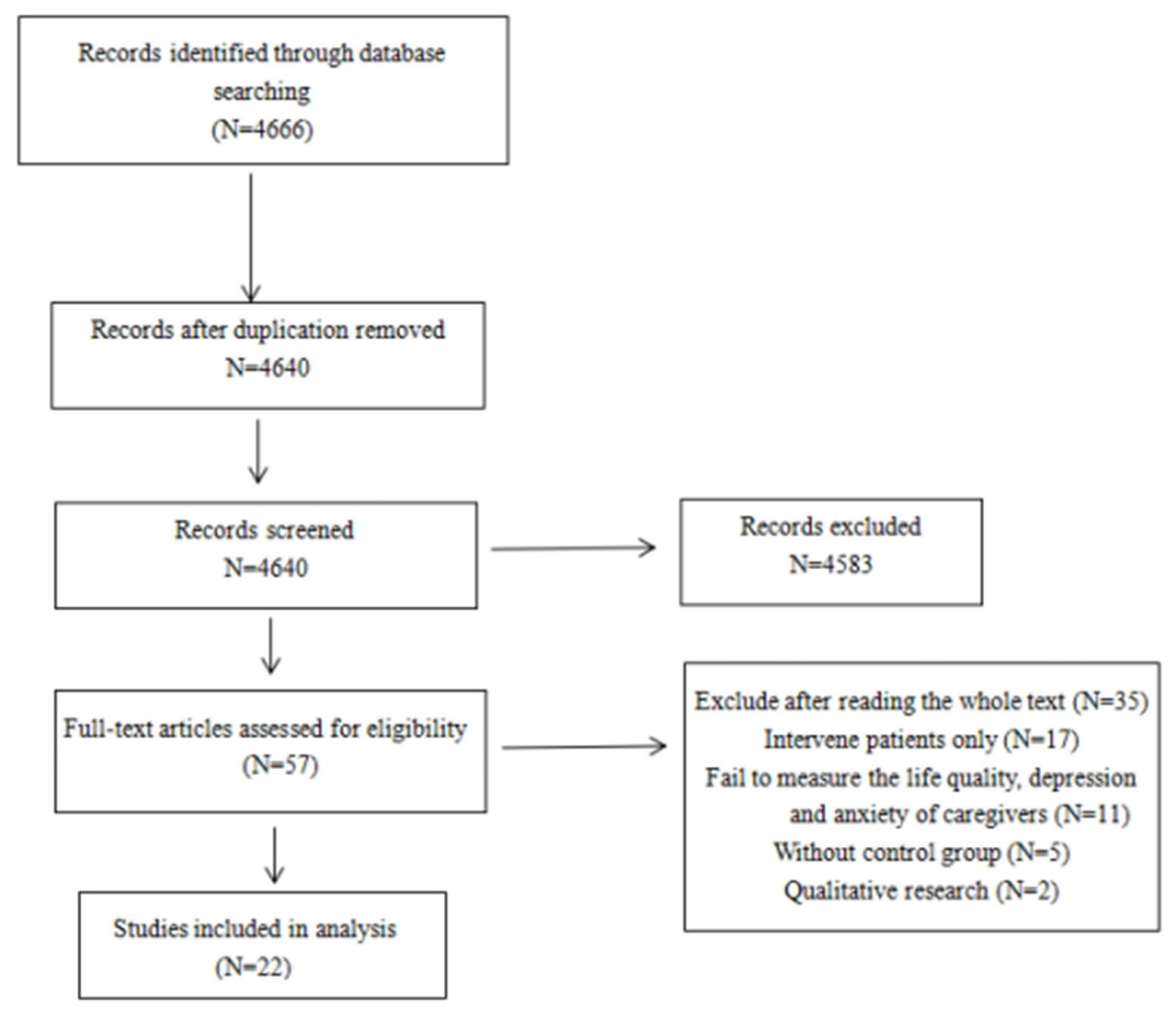
**Final process of inclusion and exclusion of studies for literature review**.

Firstly, the researcher preliminarily screened 4,666 relative studies in the literature and 26 repeated studies, and 4,583 articles' titles or abstracts were excluded from further analysis. Then, the researcher screened the remaining 57 works by examining the whole text, and finally included 22 works for comprehensive review. All of 22 studies reported changes of cancer caregivers' life quality, depression and anxiety after the interventions (see Figure 1).

### Caregiver characteristics

A total sample of 3,604 cancer caregivers enrolled at baseline and the number of caregivers in each study ranged from 20 to 484 (see Table 1). Among all studies, the overall participant attrition rate was 23.7% and study attrition rate varied from 0% to 65.8%. The reasons for attrition include the worsening or death of patients, the business of cancer caregivers. Of the 22 identified studies, three did not provide sufficient data to compute an effective size (Meyers et al., 2011; Porter et al., 2011; Leow et al., 2015).

**Table 1:** **Psychosocial intervention of cancer caregivers**.

**Author, year**	**N**	**Relation to patients**	**Age (M/SD)**	**Cancer type**	**Intervention**	**Follow-up measurement**	**Planned outcomes**
Badger et al., 2011	70	Partner	61.13 (10.9)	Prostate	TG: dyadic telephone interpersonal counseling CG:Education	8 weeks 16 weeks	Psychological well-being (depression, positive and negative affect, perceived stress) Physical well-being Social well-being Spiritual well-being
Meyers et al., 2011	476	Spouse Adult children parent Unrelated Other	61.4	Gastrointestinal Genitourinary Thoracic	TG:Paired home care guide CG:standard care	30 days 60 days 90 days 120 days 180 days	Primary outcomes: Hope, Quality of life Secondary outcome: Social Problem Solving
Lai et al., 2012	34	Spouse Parent Adult children	Nil	Breast genitourinary gastrointestinal head and neck lung	TG:music therapy by nurse CG: music therapy without nurse	Post-test	Sleep Depression Anxiety HRV
Hendrix et al., 2013	120	Spouse Adult children Other	Nil	Hematological Malignancy	TG:paired caregiver training	1 week 2 weeks 4 weeks	Primary outcome: Self-efficacy Secondary outcomes: Depression Anxiety Quality of life
					CG: individualized health education		
Porter et al., 2011	233	Spouse Adult children Sibling and friend	59.3 (12.3)	Lung	TG:Paired coping skill training CG: cancer information	Post-test 4 months	Caregiver mood (Depression, anxiety.) Caregiver strain Self-efficacy
Clark et al., 2013	131	Nil	58.7 (10.6)	Genitourinary gastrointestinal head and neck lung	TG:paired structured CBT training CG:standard care	4 weeks 27 weeks	Quality of life
Sherwood et al., 2012	225	Spouse Other	53.8 (12.7)	Nil	TG: dyadic symptom management and problem solving by nurse CG: symptom management by coach	10 weeks 16 weeks	Depressive symptom Caregiver Burden Mastery Caregiver-patient communication
Belgacem et al., 2013	67	Spouse Adult children Sibling Parent Friend	56.6(20.4)	Hematology oncology	TG: paired caregiver education program CG:standard care	1–3 months	Quality of life Caregiver burden
Boele et al., 2013	56	Nil	50.77(11.47)	High-grade glioma	TG:individualized psycho-education CG:standard care	2 months 4 months 6 months 8 months	Quality of life Caregiver mastery(CM)
Fegg et al., 2013	133	Partner Parent Adult children Other	54.3	Gynecological Lung Breast Brain	TG:existential behavioral therapy CG:usual service	Post-test 3 months 12 months	Primary outcomes: Somatization Anxiety Depression Life satisfaction Quality of life
Ledderer et al., 2013	42	Spouse Other	Nil	Gynecological lung	TG: multimodal psychosocial rehabilitation CG: usual service		Quality of life
McLean et al., 2013	42	Spouse	48.82 (13.38)	End-stage cancer	TG: paired emotion-focused therapy CG:standard care	Post -test 3 months	Primary outcome: Marital Functioning Secondary outcomes: Depression Hopelessness Empathetic caregiving Caregiver burden
Mitchell et al., 2013	329	Spouse Parent Adult children Sibling Other	58.3 (12.6)	Advanced cancer	TG:counseling based on needs CG:usual service	1 months 3 months 6 months	Unmet needs Anxiety Depression Health-related quality of life
Northouse et al., 2013	484	Spouse Other	56.7 (12.6)	Advanced Breast, Lung Colorectal Prostate cancer	TG:paired FOCUS programme CG:standard care	3 months 6 months	Quality of life
Barrera et al., 2014	67	Parent	39.21 (8.67)	Leukemia Lymphoma Brain Bone	TG:PAT assessment CG:usual service	6 months	Pediatric Quality of life Behavioral Assessment Parental Anxiety
DuBenske et al., 2014	234	Spouse Other	56.56 (12.86)	Advanced Lung Cancer	TG:e-learning of CHESS CG:standard care, website information	2 months 4 months 6 months 8 months	Primary outcomes: Disruptiveness Burden Negative mood (Depression, Anxiety, Anger) Secondary outcome:
Badr et al., 2015	39	Spouse Adult children Other	51.10 (10.24)	Advanced lung cancer	TG:paired intervention by providing nursing information plus telephone counseling CG:standard palliative care	8 weeks	Primary outcome: Psychological functioning (depression, anxiety) Secondary outcomes: Autonomy Competence Relatedness
Dionne-Odom et al., 2015	122	Nil	60	Palliative care	TG:structured telephone grief counseling 1 months after bereavement CG: grief counseling 3 months after bereavement		Quality of life Depression Caregiver burden
Leow et al., 2015	80	Nil	Nil	Advanced cancer	TG:psychological education program CG:standard care		Quality of life Depression Self-efficacy Social support
Sun et al., 2015	354	Nil	57.54 (14.31)	Palliative care of lung cancer	TG:palliative care CG:standard care	7 weeks 12 weeks	Quality of life Psychological distress Caregiver burden Caregiver skill preparedness
Hendrix et al., 2016	138	Spouse Adult children Parent Other	56.2 (12.7)	Nil	TG: paired intervention of nursing training CG:standard care	Post-test 2 weeks 4 weeks	Primary outcomes: Self-efficacy on stress management and symptom management Preparedness Secondary outcome: Psychological well-being (Depression Anxiety Burden)
Shaw et al., 2016	128	Spouse Adult children Parent Sibling Friend Other	55.7 (14.9)	Prognosis Gastrointestinal cancer	TG: family connect individualized intervention CG:standard care	3 months 6 months	Quality of life Unmet needs Burden Distress

*TG, treatment group; CG, control group*.

The main caregivers was identified as a spouse or adult child, parent, brother and sister, friend or significant other. Most of the main caregivers are spouses (69.68%). There are two studies specifically focusing on spouses (McLean et al., 2013) or partners (Badger et al., 2011; van de Hurk et al., 2015), one study focuses on young parents (Barrera et al., 2014). The mean age of the caregivers was 55.34. Most of the caregivers were women, accounting for 65.02%. Two studies were specifically for female caregivers' intervention (Badger et al., 2011; Lai et al., 2012). In terms of cancer types, there were four studies specifically for lung cancer patients (Badger et al., 2011; Porter et al., 2011; DuBenske et al., 2014; Badr et al., 2015; van de Hurk et al., 2015), one for glioma (Boele et al., 2013), one for hematologic malignant tumor (Hendrix et al., 2013). Furthermore, there were 15 articles focused on specific cancer stage of patients: newly diagnosed cancer (Barrera et al., 2014); advanced lung cancer (DuBenske et al., 2014; Badr et al., 2015); high-grade glioma (Boele et al., 2013); hematologic malignant tumor patients (Hendrix et al., 2013). The rest of the research consists of patients with different types of cancer.

### Intervention format and characteristics

In terms of intervention types, there were 11 studies of families-patients paired skills training (Badger et al., 2011; Meyers et al., 2011; Porter et al., 2011; Belgacem et al., 2013; Clark et al., 2013; Fegg et al., 2013; Hendrix et al., 2013, 2016; McLean et al., 2013; Northouse et al., 2013). What's more, two studies evaluated family demands (Mitchell et al., 2013; Barrera et al., 2014) and seven studies conducted individual skills training to caregivers (Belgacem et al., 2013; Boele et al., 2013; Dionne-Odom et al., 2015; Leow et al., 2015; Sun et al., 2015; Shaw et al., 2016). Additionally, there was another two interventions studies focused on couple-based intervention of marital relationships (McLean et al., 2013) and group skills training (Fegg et al., 2013).

With regard to the mode of intervention, it consisted of family connect intervention, including the assessment of caregivers' needs, family relationship maintenance, self-care of caregivers and so on (Shaw et al., 2016); FOCUS programme for promotion of family relationship and positive attitude, efficiency of solving problems, reduction of the uncertainty and symptom management (Northouse et al., 2013); intervention models based on self-determination theory (SDT), teaching self-care of caregivers, the patients' symptom management, relationship maintenance strategies and cognitive behavioral strategies to cope with depression and anxiety (Badr et al., 2015); using CBT mode to increase caregivers' care capacity (Boele et al., 2013; Clark et al., 2013); paired intervention of emotional focus therapy (EFT) (McLean et al., 2013), facilitation of communication and mutual support between patients and family members (Ledderer et al., 2013); education of symptom management and emotional stress reduction intervention (Porter et al., 2011; Lai et al., 2012; Sherwood et al., 2012; Belgacem et al., 2013; Hendrix et al., 2013, 2016; Leow et al., 2015; Sun et al., 2015). While symptom management referred to providing information about prevention of infection, control of pain, maintenance of nutrition, stress reduction intervention covered deep breathing, muscle relaxation and imagination of pleasure; comprehensive health enhancement support system (CHESS) model, which was a system for elevating the skills of family members of cancer patients based on a network, and it contained all kinds of information about cancers, served as a platform for communication and support between different family members and also provided feedback to family members (DuBenske et al., 2014); existential behavior therapy (EBT), including meditation, search for meaning, self-care and stress management, seeking personal value again and farewell (Fegg et al., 2013); telephone interpersonal counseling (TIP-C), which was based on the theory of interpersonal therapy plus cancer education (Badger et al., 2011); supportive talks and residential rehabilitation course (Ledderer et al., 2013) needs assessment tools like NAT-C and PAT (Mitchell et al., 2013; Barrera et al., 2014); problem behavior coping model (COPE) dealing with problems like body and mind control of patients, resources and management (Meyers et al., 2011); grief counseling (Dionne-Odom et al., 2015).

First of all, intervention format of included studies could be divided into three aspects: families-patients paired group intervention, caregivers' individual intervention and group intervention. Among them, 11 studies offered interventions to both patients and their family caregiver, 12 interventions were delivered solely to the caregiver and one provided group intervention. In terms of time and frequency of the intervention, most of them were comparatively regular, about once a week or biweekly, and the time span ranged from one and a half months to 2 years. In addition, relatively short interventions lasted 1–2 h or 2–3 h were also provided (Hendrix et al., 2013, 2016).

Regarding the practitioner of the interventions, eight were done by nurses (Porter et al., 2011; Lai et al., 2012; Sherwood et al., 2012; Belgacem et al., 2013; Hendrix et al., 2013, 2016; Ledderer et al., 2013; Northouse et al., 2013); six were conducted by professional psychologists, oncologists, behavioral therapists and trained health educators (Meyers et al., 2011; Boele et al., 2013; Fegg et al., 2013; McLean et al., 2013; Badr et al., 2015; Shaw et al., 2016); four were performed by mufti-disciplinary teams including professionals like psychotherapists, specialists and physical therapists (Clark et al., 2013; Mitchell et al., 2013; Barrera et al., 2014; Sun et al., 2015), one were led by social workers or nurses (Badger et al., 2011) and one was operated by family members through online distance learning (DuBenske et al., 2014).

### Outcome measures

In terms of the subject of research, eight studies measured quality of life (Meyers et al., 2011; Boele et al., 2013; Clark et al., 2013; Fegg et al., 2013; Ledderer et al., 2013; Northouse et al., 2013; Sun et al., 2015; Shaw et al., 2016), two studies examined depression (Sherwood et al., 2012; McLean et al., 2013), and another two studies took anxiety as the sole outcome variable (Porter et al., 2011; Barrera et al., 2014). In addition, three measured quality of life, depression and anxiety simultaneously (Fegg et al., 2013; Hendrix et al., 2013; Mitchell et al., 2013). Four examined both anxiety and depression (Lai et al., 2012; DuBenske et al., 2014; Badr et al., 2015; Hendrix et al., 2016), three took both quality of life and depression as the outcomes (Badger et al., 2011; Dionne-Odom et al., 2015; Leow et al., 2015).

Moreover, there was a lack of consistency in the measurement tools used to assess QoL, depression and anxiety. The measurement tool of QoL varied from the Caregiver Quality of Life Index-Cancer (*n* = 3); City of Hope QoL (*n* = 2); SF-12v2 (*n* = 2); SF-36 Health Survey (*n* = 1); the General Functional Assessment of Cancer Therapy Scale (FACT) (*n* = 1); Health Related Quality of Life (HRQOL) (*n* = 1); WHOQOL-BREF (*n* = 1); and Satisfaction with Life Scale (SWLS) (*n* = 1). In 10 of the 14 studies, a statistically significant improvement in general QoL was identified. In the remaining four studies, no significant change in QoL was observed. Of the nine studies where effective sizes could be calculated, three studies had effective size suggesting a small to medium effect of interventions on QoL outcomes (ranging from 0.27–0.62) (Cohen, 1988). In addition, six studies measured follow-up effect of intervention, and the time point varied from 4 weeks to 12 months. Four studies demonstrated a nil effect of treatment on QoL with Cohen d ranging from 0.07 to 0.18. The remaining two studies had effect sizes suggesting a small (0.21–0.25) and medium (0.43–0.52) effect of treatment on QoL respectively.

With respect to anxiety, the measurement tools varied from Profile of Mood State (POMS-B) (*n* = 3); Hospital Anxiety and Depression Scale (HADS) (*n* = 2); State Trait Anxiety Inventory (STAI) (*n* = 2); Brief Symptom Inventory (BSI) (*n* = 1); Patient Reported Outcomes Measurement Information System (PROMIS) (*n* = 1). In seven of the nine studies, a statistically significant improvement in anxiety was identified. In the remaining two studies, no significant change in anxiety was observed. Of the six studies where effective sizes could be calculated, two studies had effective size suggesting nil to small effect of interventions on anxiety (ranging from 0.11 to 0.41) and another two studies had high effect of interventions on anxiety with estimated d ranging from 1.12 to 1.3 (Cohen, 1988). Moreover, two studies measured follow-up effect of intervention, and the time point varied from 1 to 8 months. Both studies demonstrated a nil to small effect of treatment on anxiety with Cohen d ranging from 0.04 to 0.44.

As for depression, measurement tools consisted of Center for Epidemiology Studies-Depression Scale (CES-D) (*n* = 4); Hospital Anxiety and Depression Scale (HADS) (*n* = 2); Profile of Mood State (POMS-B) (*n* = 2); Brief Symptom Inventory (BSI) (*n* = 1); Beck Depression Inventory (BDI) (*n* = 1); Patient Reported Outcomes Measurement Information System (PROMIS) (*n* = 1); and self-developed depression scale (*n* = 1). In eight of the 13 studies, a statistically significant improvement in depression was identified. In the remaining five studies, no significant change in anxiety was observed. Of the seven studies where effective sizes could be calculated, two studies had effective size suggesting small effect of interventions on anxiety (ranging from 0.27 to 0.39) and another study assessed depression demonstrating a large effective size (*d* = 1.8) (Cohen, 1988). Additionally, four studies measured follow-up effect of intervention, and the time point varied from 6 weeks to 12 months. Four studies demonstrated a nil to small effect of treatment on anxiety with Cohen d ranging from 0.04 to 0.47 (Cohen, 1988).

As was shown in Table 2, among the 21 interventions, 17 had shown effects in psychological health on at least one dimension. group training was effective in improving quality of life, easing anxiety and depression (Fegg et al., 2013). Furthermore, paired-intervention of self-care skills to cancer caregivers and symptom management to patients (Badr et al., 2015) and individual training of stress coping skills (Lai et al., 2012; DuBenske et al., 2014; Leow et al., 2015) Assessment of the families' demand (Mitchell et al., 2013), education of symptom management, stress coping and problem-solving (Meyers et al., 2011; Belgacem et al., 2013; Boele et al., 2013; Northouse et al., 2013) were specifically effective in improving quality of life.

**Table 2:** **Significance of effectiveness of included studies**.

**Author, year**	**Risk of bias**	**Theoretical foundation**	**Intervention strategies**	**Quality of life**	**Anxiety**	**Depression**
Badger et al., 2011	Medium	Social support and interpersonal theories	TIP-C: Symptom management Emotional expression Interpersonal communication and relationships Social support and cancer information	Y 6 weeks (*d* = −0.21) 12 months (*d* = −0.25)		Y 6 weeks (*d* = 0.13) 12 months (*d* = 0.20)
Meyers et al., 2011	Medium	Cognitive-behavioral problem-solving educational intervention	Teach creativity, optimism, planning, expert information attitude (COPE) problem-solving model address identified problems	Y (no data provided)		
Hendrix et al., 2013	Low	Self-efficacy theory	Prevention of infection Pain control Maintenance of nutrition adequate elimination	N	N	N
Lai et al., 2012	Medium	Psycho-physiological theory	Music intervention		Y (*d* = 1.12)	Y (*d* = − 0.27)
Porter et al., 2011	Low	Cognitive-behavioral principles	Alter cancer-relevant thoughts, emotions, and behaviors through training in coping skills, such as relaxation, cognitive restructuring, problem-solving		Y (no data provided)	N
Clark et al., 2013	Medium	Focused on specific strategies to address all 5 quality of life domains	Multidisciplinary intervention Education, cognitive behavioral strategies for coping with cancer, open discussion, support, deep breathing or guided imaginary relaxation segment	Y 4 weeks (*d* = 0.43)		
Sherwood et al., 2012	Medium	Nil	Symptom management and problem-solving intervention Help patients implement self-care Providing information on symptom management Counseling on how to engage caregivers to communicate with others			N
Belgacem et al., 2013	Low	Nil	Caregiver educational program Meal support Nursing care Welfare care Symptom management	Y Post-test (*d* = 0.62)		
Boele et al., 2013	Medium	Cognitive behavioral therapy (CBT)	Document history and functioning of patient and caregiver Introduce rational of CBT Discuss selective topics of informal caregivers	Y 4 months (*d* = − 0.18) 6 months (*d* = − 0.07) 8 months (*d* = 0.15)		
Fegg et al., 2013	Low	Existential Behavioral Therapy(EBT)	Introduction of mindfulness Activating resources and finding meaning Self-care and stress management Personal values for re-orientation	Y Post-Test (*d* = 0.43) 3 months (*d* = 0.16) 12 months (*d* = 0.52)	Y Post-test (*d* = −0.11)	Y Post-test (*d* = −0.13) 12 months (*d* = −0.27)
McLean et al., 2013	Medium	Emotional Focused Therapy (EFT)	Increase more engagement and flexible response patterns Strengthen the attachment bond Mitigate grief and loss			Y Post-test (*d* = 0.42) 3 months (*d* = 0.47)
Mitchell et al., 2013	Low	Needs Assessment Tool-Carers (NAT-C) NAT-C-guided consultations	Assess carers' unmet needs across informational, physical, psychological, spiritual, existential, social, financial and legal domains Provide links to evidence-based information, resources and services help address identified problems	Y 6 months (*d* = −0.13)	Y 1 months (*d* = 0.34) 6 months (*d* = −0.44)	N
Northouse et al., 2013	Low	Stress-coping theory	FOCUS : family involvement optimistic attitude coping effectiveness uncertainty reduction symptom management	Y 3 months (*d* = 0.15) 6 months (*d* = 0.14)		
Barrera et al., 2014	Medium	Conceptual model of pediatric psychosocial preventive health	Providing a summary of family psychosocial risk information to the medical team of a child		Y Post-test (*d* = −0.41)	
DuBenske et al., 2014	Low	Model of coping self-efficacy	CHESS Web-based model to help users appraise the controllability of cancer-related stressors and improve cognitive, behavioral and supportive coping skills		Y 2 months (*d* = 0.04) 4 months (*d* = 0.12) 6 months (*d* = 0.41) 8 months (*d* = 0.24)	Y 2 months (*d* = 0.04) 4 months (*d* = 0.12) 6 months (*d* = 0.41) 8 months (*d* = 0.24)
Badr et al., 2015	Low	Self-determination theory	Teach skills of self-care and managing symptoms Support autonomy Improve interpersonal connections		Y Post-test (*d* = −1.3)	Y Post-test (*d* = −1.8)
Dionne-Odom et al., 2015	Medium	Educate, Nurture, Advise Before Life Ends(ENABLE) Model	Problem solving using the framework of creativity, optimism, planning, expert information (COPE) attitude	N		Y Post-test (*d* = −0.39)
Leow et al., 2015	Medium	Psycho-education intervention		Y (no data provided)		Y (no data provided)
Sun et al., 2015	Medium	Interdisciplinary palliative care intervention	Baseline assessment Personalized palliative care plan, Make recommendations on symptom management and supportive-care referrals for patients and supportive referrals and available community resources	Y Post-test (*d* = 0.27)		
Hendrix et al., 2016	Low	Nil	Enhanced-CT Symptom and stress management Prevention of infection Management of fatigue Pain control Maintenance of nutrition Deep breathing Progressive muscle relaxation Pleasant imagery		N	N
Shaw et al., 2016	Medium	Family connect telephone Intervention	Assessment and intervention of patient care Maintaining family relationship Emotional and physical self-care Information and practice needs	N		

*Y, effective; N, not effective; D, Cohen' d*.

With regard to the practitioners of the interventions, six of the interventions conducted by psychological consultants, researchers, health educators and oncologists were effective (Badger et al., 2011; Meyers et al., 2011; Boele et al., 2013; Fegg et al., 2013; McLean et al., 2013; Badr et al., 2015), four of the interventions led by nurses were effective (Porter et al., 2011; Belgacem et al., 2013; Northouse et al., 2013; Barrera et al., 2014), one of the interventions carried out by social workers or nurses was effective (Barrera et al., 2014). In addition, four of the interventions operated by the multidisciplinary teams composed of oncologists, radiotherapists, psychological consultants, physiotherapists and physical therapists, physicians and nurses and social workers were effective (Clark et al., 2013; Mitchell et al., 2013; Barrera et al., 2014; Sun et al., 2015). Moreover, self-help e-learning by caregivers was also proved to be effective (DuBenske et al., 2014).

### Methodological quality

Among current interventions, 91% applied RCT, two of which were quasi-experimental design (DuBenske et al., 2014; Sun et al., 2015). In terms of the measures of comparative interventions, there were three kinds of compared groups: 13 interventions adopted the normal mode, four adopted controlling, four were used for information provision and one is made pending (Dionne-Odom et al., 2015). Furthermore, from 2 weeks after the baseline assessment to 1 year after the baseline assessment, 11 interventions got two follow-up visits, and five got three to four; one included only pre and post-tests, and one got five follow-up visits. Additionally, eight interventions included post-tests while the other 14 interventions included none and only collect data from the follow-up visits.

Crocharane, the ROB assessment tool, was used to evaluate the overall ROB. The result showed that only one intervention was assessed with high ROB (Ledderer et al., 2013), while 12 interventions had middle ROB and nine had low ROB (see Table 2). The one with high ROB would not be discussed further.

## Discussion

This research systematically reviewed 22 pieces of experiments on interventions for cancer caregivers regarding of their quality of life, depression and anxiety, 19 of which adopted the method of a randomized controlled trial. Comparing to the reviews conducted by Harding and Higginson (2003) and Waldron et al. (2013), in the recent 6 years, both the quality and quantity of research on this topic had improved considerably (Harding and Higginson, 2003; Waldron et al., 2013).

There were three types of interventions among the experiments targeting cancer caregivers, including individual training, group training and paired-intervention. Paired-intervention had recently become a new trend and shown a sizable effect on improving quality of life (Belgacem et al., 2013) as well as alleviating depression and anxiety at the same time (Badr et al., 2015). Performing targeted interventions to patients and caregivers, along with instructing on the communication and emotional support between patients and caregivers, had an apparent effect on patients and their caregivers. Yet, it should be pointed out that paired-intervention did not necessarily have to take place through the whole intervention process. Some research showed that some caregivers or patients tend not to receive all the counseling together with the other party. Sometimes, they wished to be with the consultant by themselves. In this way, they could better explain their feelings and worries without influencing each other. The traditional way of individual intervention on caregivers also proved to be effective. Intervening face-to-face with the caregivers led to better-directing contents on handling the symptoms, relieving emotional burdens and pressure and self-adjustment—very practical and effective for the caregivers.

In addition, the research showed that an evaluation of caregivers' needs could help with their quality of life and depression, the result of which was similar to those reported by other systematic review (Waldron et al., 2013). For caregivers, receiving an evaluation meant that they could openly talk about their worries and problems, and their words would be listened to and understood by the professionals, which could reduce the stress of caregivers. Concerning the conductors of interventions, experiments showed that nurses as well as psychotherapists, health educators and oncologists played the main role in implementing the psycho-social intervention for cancer caregivers while social workers were not further involved. Even in interventions conducted by multidisciplinary team, social workers were only mentioned in one study (Barrera et al., 2014), which implies that the role of social worker in psychosocial intervention had not been acknowledged and given full play.

In terms of the contents of interventions, the previous systematic review concluded that intervention focusing on the communication and education of patients and their caregivers could improve caregivers' quality of life (Waldron et al., 2013). This research provided further evidence to support the result. Providing information on cancer education and on practicing a healthy life could help patients and their caregivers in raising their quality of life. In addition, this review found that COPE, existential treatment and social psychological evaluation on patient families were also effective in improving their quality of life.

Regarding the alleviation of depression and anxiety of cancer caregivers, research showed that 30 min of music treatment can release caregivers' anxiety and soothe their emotions. Moreover, the treatment effect could be better with the presence of nurses, which created a warm and comforting atmosphere. At the same time, this review found that more attention should be paid to personalized evaluation and intervention of care needs and that hospice care at the beginning stage of cancer could relieve the caregivers of depression.

In addition, this review found that rather complicated systematic intervention did not show greater advantage over coping intervention or health education (Porter et al., 2011). Thus, further research on the effective factor among intervention modes was needed. What do we actually need—a complicated intervention form or a more distinctive intervention factor? Maybe it is the time spent and the attention paid to caregivers that are effective rather the intervention itself. Furthermore, the effectiveness of many intervention studies was not sustainable. The reasons could be that simple intervention modes failed to satisfy the needs of caregivers according to various types and phases of cancer. As a result, it was necessary to add focusing methods into the intervention mode, providing customized service and support. Furthermore, in randomized controlled trials, a high drop-out rate was a possible reason for the ineffectiveness of intervention (Shaw et al., 2016). Thus, measures on maintaining the initiative of the subjects could better ensure the effect of intervention.

### Limitations and future research directions

This review took only three indexes into consideration, which are caregivers' quality of life, degree of depression and degree of anxiety. Thus, there could be selection bias. In addition, quality of life is a broad concept without consistent definition, and no one definition was used in current systematic review, which limits the generalizability of the results of this review. At the same time, the cancer types and stages as well as intervention mode and length varied widely in this review, making it difficult to compare the effectiveness of interventions across included studies. Furthermore, this study only reviewed research in English and Chinese, which could also limit the generalizability of the results. Reviewing only the quantitative research could also increase selection bias. Furthermore, most research was conducted in developed countries such as European countries and the United States. Due to the differences in both culture and policy, whether or how the conclusions could be extended in other culture contexts needs further confirmation.

## Author contributions

FF is the primary researcher of this study, responsible for analyzing data and writing paper. HZ is the second author, responsible for analyzing data and writing paper. FT is the third author, responsible for supervising the data analysis and paper writing. IC is the fourth author, responsible for supervising the process of paper writing.

### Conflict of interest statement

The authors declare that the research was conducted in the absence of any commercial or financial relationships that could be construed as a potential conflict of interest.
